# Supplementary material for: Differences in emergency hospitalization trauma patients during and after the COVID-19 pandemic
Source: PLoS One. 2024 Dec 18;19(12):e0315416. doi: 10.1371/journal.pone.0315416 (PMC11654972; doi:10.1371/journal.pone.0315416)
Supplement: Attachment — Submitted filename: Response to Reviewers 2.docx [file pone.0315416.s004.docx]

Shenzhen Third People’s Hospital, Second Hospital Affiliated to Southern University of Science and Technology

29 Bulan Road, Longgang District, Shenzhen, Guangdong, China

Dear Editor,

I am very sorry for my poor English, and it is the first time for me to submit a paper to *PLOS ONE*, so I face a lot of difficulties. Thank you very much for your patience, and I will work harder. When I upload my data to Dryad, it indicates that I have 10 alerts, which is very tedious for me, so I want to modify it again. I look forward to your forgiveness.

Thank you for your letter and for the reviewers' comments concerning our manuscript entitled *Differences in Emergency Hospitalization Trauma Patients (PONE-D-24-16756R1)*.Those comments are all valuable and very helpful for revising and improving our paper,as well as the important guiding significance to our researches. We have studied comments carefully and lave made corrections which we hope to meet with *PLOS ONE*'s approval.We highlighted the changes in yellow.

Response:

Regarding data, for this submission, I choose that some restrictions will apply and all relevant data are included within the manuscript and its Supporting Information files.

Thank you very much for your attention and time.**Please do not hesitate to contact us if there are any question,I will reply as soon as possible**.Thanks again to the reviewers and editors for your hard work!Best wishes to you!

Yours sincerely

Gengwei Zhang

November 7, 2024
